# High expression of *PCOLCE* gene indicate poor prognosis in patients and are associated with immune infiltration in glioma

**DOI:** 10.1038/s41598-023-30413-5

**Published:** 2023-03-07

**Authors:** Qingbao Guo, Xin Gao, Jingjie Li, Yukun Liu, Jiayu Liu, Hui Yang, Meng Cui, Meng Zhang, Lian Duan, Xiaodong Ma

**Affiliations:** 1 Medical School of Chinese PLA, Beijing, China; 2 Department of Neurosurgery, The First Medical Centre, Chinese PLA General Hospital, Beijing, China

**Keywords:** Computational biology and bioinformatics, Genetics, Immunology, Neuroscience

## Abstract

The procollagen C-protease enhancer (*PCOLCE*) has been identified to influence tumor growth and metastasis in multiple cancers. However, the relationship between *PCOLCE* activity and the progression of gliomas remains largely unknown. Glioma RNA-seq data were derived from the Chinese Glioma Genome Atlas (CGGA) and The Cancer Genome Atlas databases for analysis. Kaplan–Meier survival curve, clinical characterization correlation, univariate and multivariate Cox, and receiver operating characteristic curve analyses were performed to assess the prognostic role of *PCOLCE*. Gene Ontology, Kyoto Encyclopedia of Genes and Genomes, and Gene Set Enrichment Analysis were used to determine the functions or pathways associated with *PCOLCE*. The ESTIMATE and CIBERSORT algorithms, Spearman’s rank correlation analysis, and Tumor Immune Estimation Resource (TIMER) databases were used to explore the relationship between *PCOLCE* and immune infiltration. Correlation analysis between *PCOLCE*, related genes, and immune cell markers was conducted using the TIMER database. Immunophenoscore assays were performed to determine differential PCOLCE expression levels in glioma. The sensitivity of multi-drugs were determined to explore potential chemotherapeutic agents in between PCOLCE. Compared to normal brain tissue, *PCOLCE* expression was increased in glioma and correlated with shorter overall survival (OS). Furthermore, significant differences were observed in the immune scores and immune cell infiltration levels. PCOLCE is positively associated with immune checkpoints and many immune markers. Additionally, PCOLCE expression was higher in gliomas with higher IPS Z-scores in CGGA. High expression of PCOLCE increased sensitivity to multiple chemotherapy agents in CGGA (P < 0.001), and TCGA. These results suggest that *PCOLCE* significantly influences the prognosis of patients with glioma, can serve as an independent prognostic factor, and is related to tumor immunity. *PCOLCE* may be a novel immune-related target for treating gliomas. Additionally, analysis of chemosensitivity in gliomas with high PCOLCE expression may provide a promising direction for drug development.

## Introduction

Glioma is a primary intracranial malignant tumor with the highest incidence rate, and an annual incidence rate of 6.6 per 100,000 people in the United States^1^. Glioblastoma multiforme (GBM) is the most malignant type of intracranial cancer with the worst prognosis, accounting for approximately half of newly diagnosed gliomas, with a median survival of approximately 14–17 months in current clinical trials^2^ and 12 months in population-based studies^3^. Few successful anti-glioma drugs and immunotherapies have been approved by the US Food and Drug Administration due to the prevention of drug absorption into the brain by the blood brain barrier^3^. Temozolomide (TMZ), a DNA intercalator agent that can cross the blood–brain barrier, is the preferred chemotherapy drug for glioma; however, this isolated drug cannot be used in GBM. Studies have shown that TMZ combined with radiotherapy can significantly prolong the median survival time of GBM patients^4^, as the 2-year survival rate increased by 16% and 5-year survival rate increased from 2 to 9.8%^5,6^. However, there has been no further breakthrough in glioma treatment, especially in GBM^6,7^. Additionally, about half of the patients with GBM display poor treatment response to TMZ, possibly due to high expression of O6-methylguanine-DNA methyltransferase (MGMT), which lead to an overwhelming amount of recurrence in patients^8^. Based on the above reasons, It is still a challenge to improve the long-term survival rate of patients with glioma. Therefore, identifying new therapeutic targets based on molecular mechanisms is vital for improving patient outcomes.

The extracellular matrix (ECM), a three-dimensional with cell-free structures, exists in all tissues and is critical to life. The ECM functions as physical support for tissue integrity and resilience and is continuously remodeled to maintain dynamic tissue homeostasis^9^. The density and direction of ECM fibers also play an essential role in modulating immunocyte migration. Flexible areas of fibronectin and collagen promote T cell motility, whereas dense ECM domains prevent migration. These ECM fibers also dominate cell migration trajectories and constrain immune cell interactions with cancer cells^10^. Bone morphogenetic protein 1 (BMP-1) is a zinc metalloprotease that can result in collagen deposition by removing C-propeptide in procollagen I, II, and III in the ECM, thereby influencing immune cell function^11^.

Procollagen C-protease enhancing protein, coded by the *PCOLCE* gene, is a secretory glycoprotein that boosts the activity of procollagen C-protease and stimulates ECM remodeling^11,12^. Moreover, Procollagen C-protease enhancing protein binds to the C-propeptide of type III procollagen via a CUB domain and heparin sulfate via an NTR domain. This binding enhances BMP-1 activity and maturation of collagen precursors, thereby affecting immune cell function^13,14^. It has been reported that dysregulation of Procollagen C-protease enhancing protein is found in various diseases. For example, high *PCOLCE* levels are implicated in muscle and liver fibrosis^15^. Low *PCOLCE* expression results in insufficient corneal repair^16^. Additionally, *PCOLCE* levels are elevated in human osteosarcoma tissues compared with adjacent non-cancerous tissues, and play a vital role in facilitating lung metastasis of osteosarcoma^17^. PCOLCE has also been shown to be a marker of immune infiltration in gastric cancer^18^.

Although several studies have probed the role of PCOLCE in different cancers and can also be used as a prognostic marker for gastric cancer, little has been reported on the role of PCOLCE in glioma. In this study, we systematically analyzed the role of PCOLCE using TCGA and CGGA databases, two of the largest glioma cohorts, and investigated its association with glioma prognosis and immune infiltration as well as sensitivity to multiple chemotherapy agents.

## Materials and methods

The glioma RNA-seq data (including a total of 701 cases, 169 GBM cases, and 532 low-grade glioma (LGG) cases) were downloaded from TCGA website (http://www.tcga.org/), and clinical data (669 cases, GBM + LGG) were downloaded from the GlioVis website (http://gliovis.bioinfo.cnio.es/). Gene expression data and the corresponding clinical information of 664 cases were obtained. RNA-seq and clinical data for patients with glioma (325 + 693 cases) were downloaded from the CGGA website (http://www.cgga.org.cn). R software (version 3.6.4) was used for data analysis and visualization. The "Limma"^19^ and "sva"^20^ packages were used for batch correction and integration. Patients with glioma in each cohort were divided into low and high PCOLCE expression groups according to the median expression level of PCOLCE.

### The expression level of *PCOLCE* in glioma and Human Protein Atlas analysis

The package "ggpubr" (https://CRAN.R-project.org/package=ggpubr) was used to analyze the different *PCOLCE* expression levels among the different glioma grades. These samples were obtained from TCGA database and compared to normal samples that were obtained from the GTEx database (https://commonfund.nih.gov/gtex). We further confirmed PCOLCE levels in normal and high-grade glioma (HGG) samples using the Human Protein Atlas (HPA) (http://www.proteinatlas.org).

### Prognosis analysis

The Kaplan–Meier curve was used for survival analysis. The "survival" (https://CRAN.R-project.org/package=survival) and "survminer" packages (https://CRAN.R-project.org/package=survivalminer) were used to analyze the data and construct Kaplan–Meier curves for the CGGA and TCGA glioma samples. Univariate and multivariate Cox analyses were performed, and receiver operating characteristic (ROC) curve survival analysis was performed using the "ROC" package (https://CRAN.R-project.org/package=survivalROC).

### Differential gene enrichment analysis

Differential analyses were performed using the "Limma" package. The "clusterProfiler"^21^ and "enrichplot" packages (https://github.com/GuangchuangYu/enrichplot) were used to perform Gene Ontology (GO) and Kyoto Encyclopedia of Genes and Genomes (KEGG) enrichment analyses of the differentially expressed genes. In addition, GSEA software was used to analyze the KEGG pathways between high and low levels of *PCOLCE*.

### PCOLCE expression level and immune infiltrates

Sample immune scores were assessed using the "ESTIMATE" package (https://R-Forge.Rproject.org/projects/estimate/) and the CIBERSORT^22^ algorithm. These were used to analyze the correlations between *PCOLCE* and 22 immune cell subsets. The Tumor Immune Estimation Resource (TIMER, https://cistrome.shinyapps.io/timer/) was used to analyze the relationship between different immune cells, prognosis in GBM and LGG, and the correlations between *PCOLCE* and immune cells. TIMER2.0, can be utilized to assess immune infiltration and systematically analyze the immune infiltration subtypes of different histopathologic types in glioma^23^. In addition, a previously published statistical deconvolution method was used to assess the abundance of tumor-infiltrating immune cells using gene expression profiles^24^.

### Correlation exploration between PCOLCE expression and immune marker sets

Previous studies have identified the gene markers of tumor-infiltrating immune cells^25,26^, including CD8^+^ T cells, monocytes, M1 macrophages, M2 macrophages, B cells, T cells (general), TAMs, follicular helper T (Tfh) cells, T-helper 1 (Th1) cells, T-helper 2 (Th2) cells, T-helper 17 (Th17) cells, Tregs, neutrophils, natural killer (NK) cells, dendritic cells (DCs), and exhausted T cells.

### Immunophenoscore (IPS) assays and investigating the sensitivity of multi-drugs

Immunophenoscore (IPS) assays were performed using the "ggplot2, grid, gridExtra" package to identify the different *PCOLCE* expression levels in glioma. The sensitivity of multi-drugs were determined utilizing the "oncoPredict, data.table, gtools, reshape2, ggpubr, limma" packages to explore potential chemotherapeutic agents in up- or down-regulated PCOLCE expression.

### Statistical analysis

Wilcoxon signed-rank test and Student's t-test were used for statistical analysis of high- and low-expressing groups. Spearman's rank correlation coefficient and statistical significance were used to evaluate gene expression correlation. The correlation strength was determined according to the following guide for the absolute value: 0.30–0.40 “moderate”, 0.40–0.50 “strong”. Significance was defined at ****P < 0.0001, ***P < 0.001, **P < 0.01, *P < 0.05. The package "ggplot2” was used to visualize the results.

## Results

### *PCOLCE* is highly expressed in glioma and is related to patient prognosis

**Figure 1:**
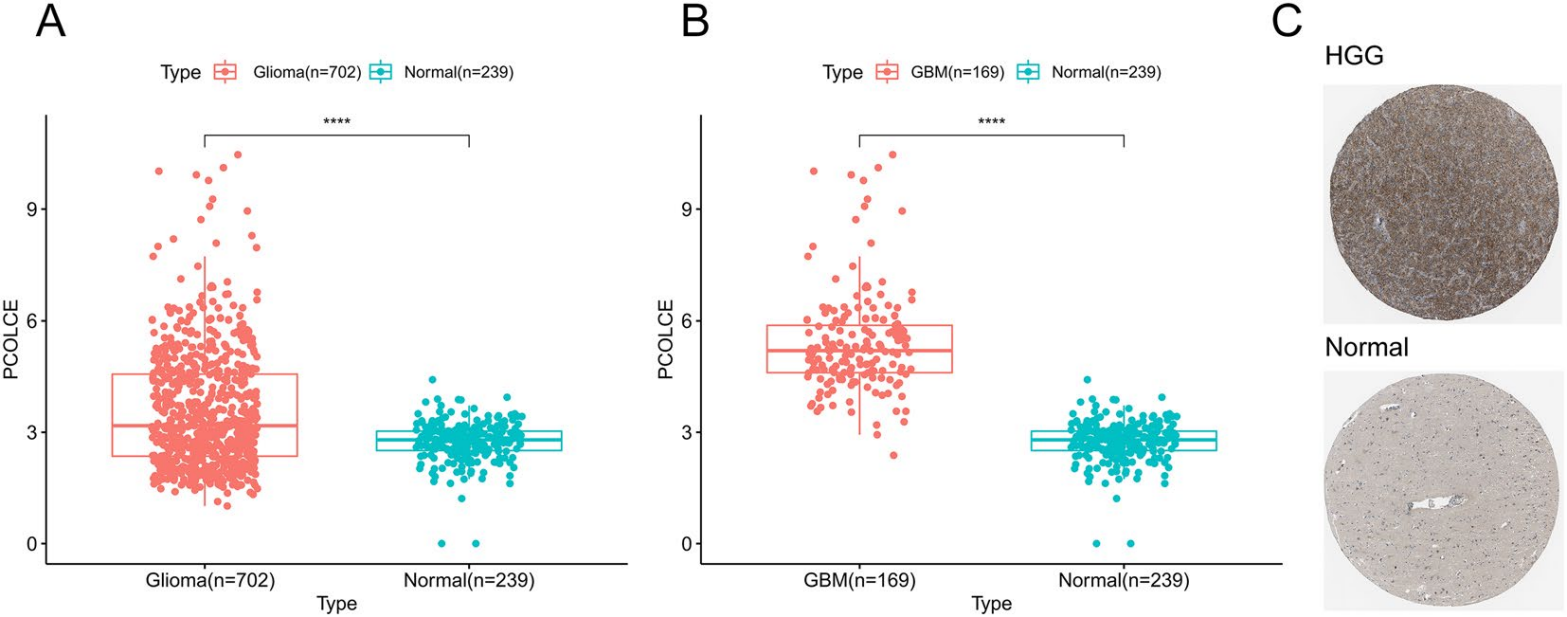
The expression level of *PCOLCE* in glioma. (**A**) Expression of *PCOLCE* in glioma based on the TCGA database and normal tissues based on the GTEx database. (**B**) Expression of *PCOLCE* in GBM based on the TCGA database and normal tissues based on the GTEx database. (**C**) PCOLCE levels in high-grade glioma and normal tissues is based on the Human Protein Atlas. ****P < 0.0001.

**Figure 2:**
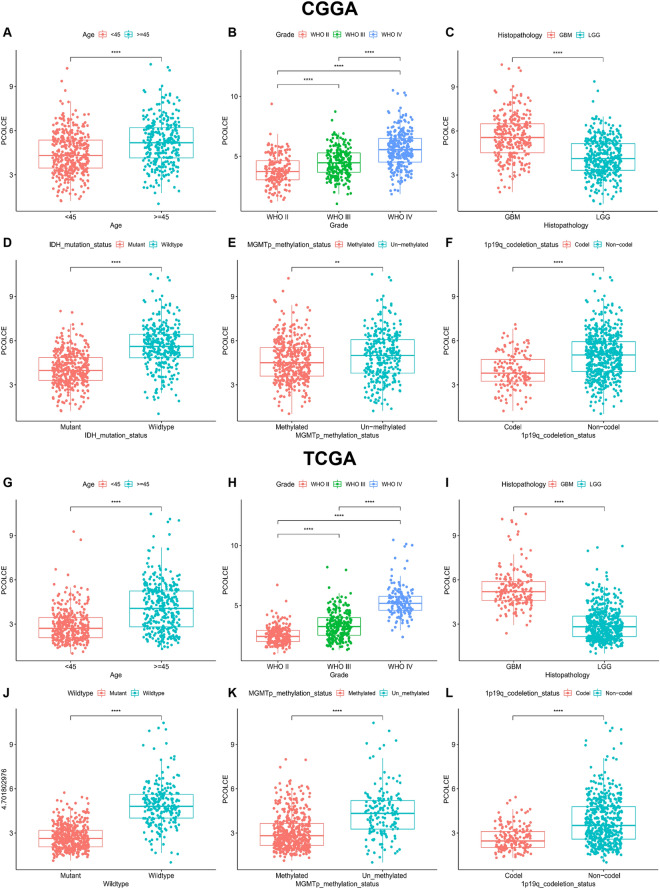
Correlation between the expression of *PCOLCE* and clinical features using the CGGA and TCGA databases. (**A**,**G**) Differential expression of *PCOLCE* was significantly associated with patient age, (**B**,**H**) WHO grade of glioma, (**C**,**I**) histopathology, (**D**,**J**) IDH mutation, (**E**,**K**) *MGMT* methylation, and (**F**,**L**) 1p19q co-deletion. *P < 0.05; **P < 0.01; ***P < 0.001; ****P < 0.0001.

*PCOLCE* was highly expressed in both glioma and GBM samples compared to normal samples (P < 0.0001, Fig. 1A,B). Similarly, PCOLCE protein levels were higher in HGG samples than in normal samples in the HPA database (Fig. 1C). Next, we analyzed the relationship between PCOLCE levels and clinicopathological features of patients with glioma. We found that *PCOLCE* expression was significantly correlated with patient age, tumor grade, histology, MGMT methylation levels, 1p19q co-deletion, and IDH mutation (P < 0.001, Fig. 2A–L) but not patient gender (P > 0.05, Supplementary Fig. 1).

**Figure 3:**
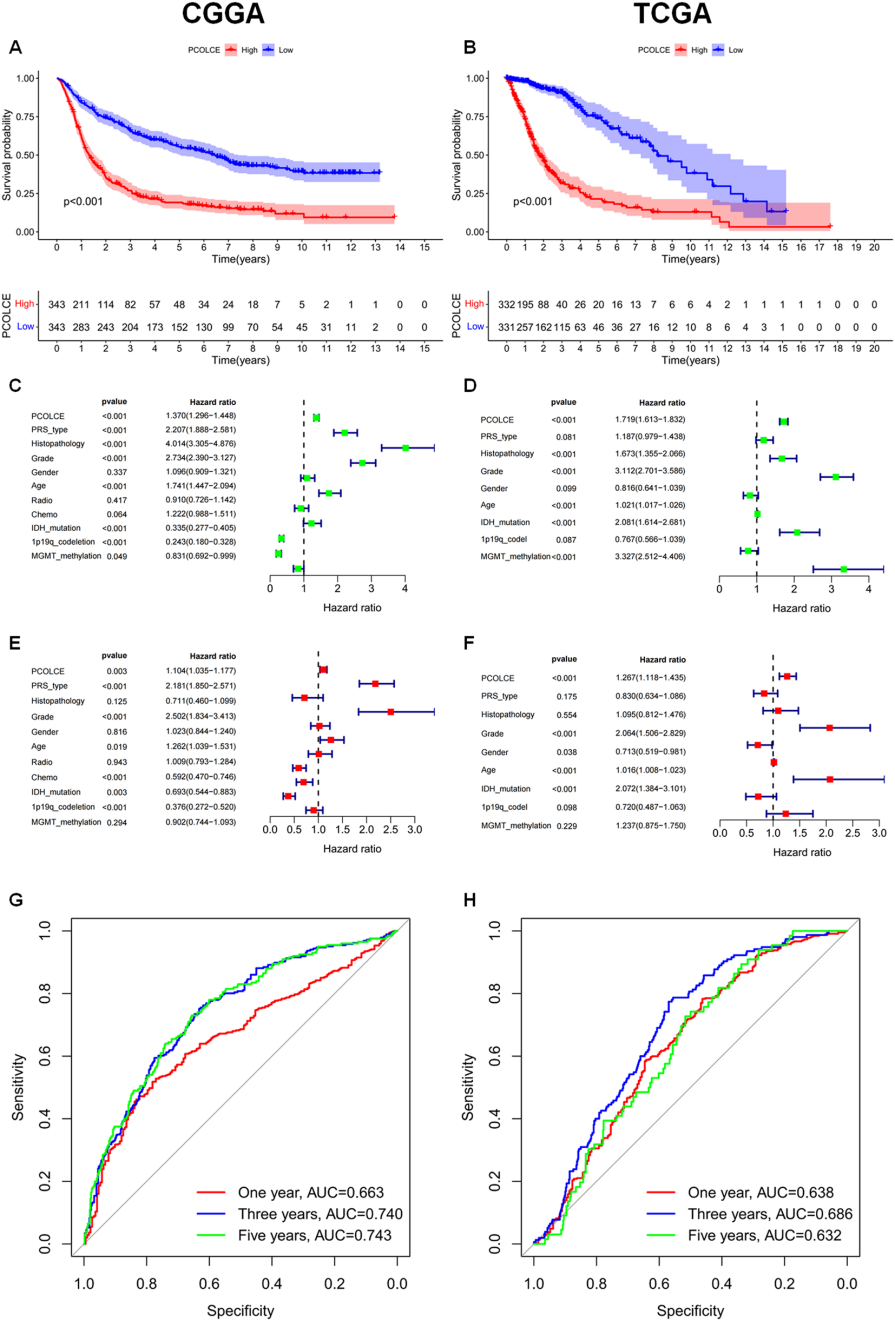
Survival analysis of PCOLCE in CGGA and TCGA patients. (**A**,**B**) Kaplan–Meier survival curves in the high and low *PCOLCE* expression groups. (**C**,**D**) Univariate Cox analysis of *PCOLCE* expression. (**E**,**F**) Multivariate Cox analysis of *PCOLCE* expression. (**G**,**H**) ROC analysis of PCOLCE for 1-, 3-, and 5-year survival.

Kaplan–Meier survival analysis indicated that high *PCOLCE* expression was significantly correlated with poor prognosis (P < 0.001, Fig. 3A,B). Furthermore, univariate Cox analysis identified *PCOLCE* as a risk factor. Before the multivariate COX analysis, we have made multicollinearity diagnosis for several independent variables. The independent variables without multicollinearity were then included in the multivariate COX analysis which revealed that *PCOLCE* was independently associated with glioma prognosis (Fig. 3C–F). Moreover, we confirmed that patient age, histology, tumor grade, chemotherapy, and IDH mutations can also affect patient prognosis (Fig. 3C–F).

In addition, the ROC curve analysis suggested that *PCOLCE* showed satisfactory performance in predicting the 1-, 3-, and 5-year survival rates of patients (all AUCs > 0.63) (Fig. 3G,H).

### Differential gene enrichment analysis between ***PCOLCE*** groups

**Figure 4:**
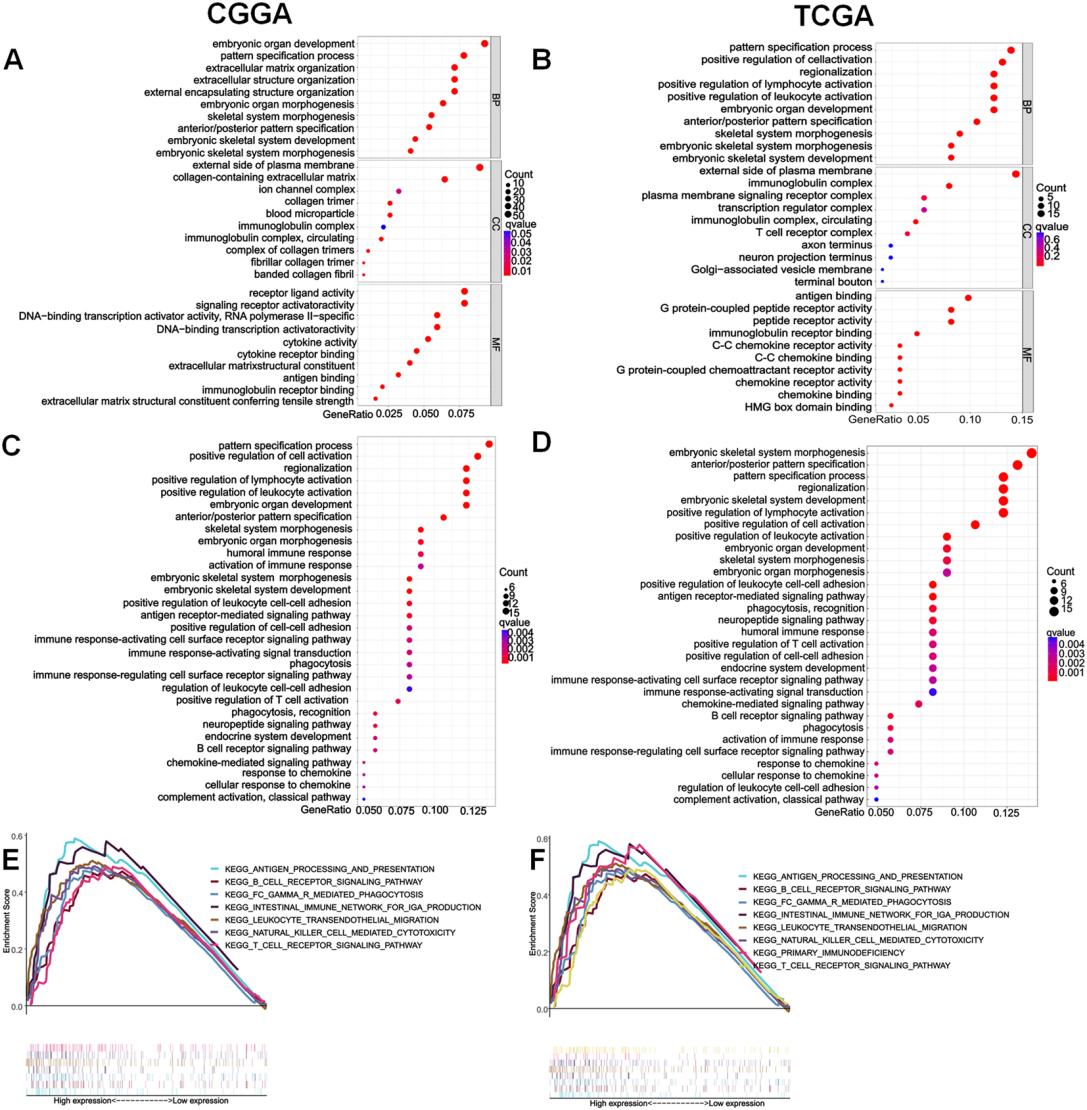
Differential gene enrichment analysis between different *PCOLCE* expression groups. (**A**,**B**) Top 10 GO terms including biological process, cellular component, and molecular function. (**C**,**D**) Top 30 KEGG pathways. (**E**,**F**) GSEA enrichment analysis revealed potential associations between *PCOLCE* and several immune-associated pathways.

We then analyzed the differential genes and produced a heatmap to display the top 50 upregulated and downregulated genes between the two groups (Supplementary Fig. 2). GO enrichment analysis of the differentially expressed genes revealed that *PCOLCE* may be significantly associated with antigen binding, immunoglobulin receptor binding, cytokine activity, cytokine receptor binding, positive regulation of lymphocyte activation, the T cell receptor complex, and other immune-related functions (Fig. 4A,B). Interestingly, KEGG analysis further suggested that *PCOLCE* may participate in immune-related and oncogenic pathways, such as the immune response-activating and regulating cell surface receptor signaling pathways, immune response-activating signal transduction, regulation of leukocyte cell–cell adhesion, positive regulation of T cell activation, and the B cell receptor signaling pathway (Fig. 4C,D). In addition, GSEA TCGA and CGGA databases indicated significant enrichment of multiple immune-related functions and pathways (Fig. 4E,F). These results suggest that *PCOLCE* may interact with the immune-associated TME. Therefore, further analysis of the relationship between *PCOLCE* and tumor immunity is necessary.

### PCOLCE may play a role in glioma through regulation of tumor immunity

**Figure 5:**
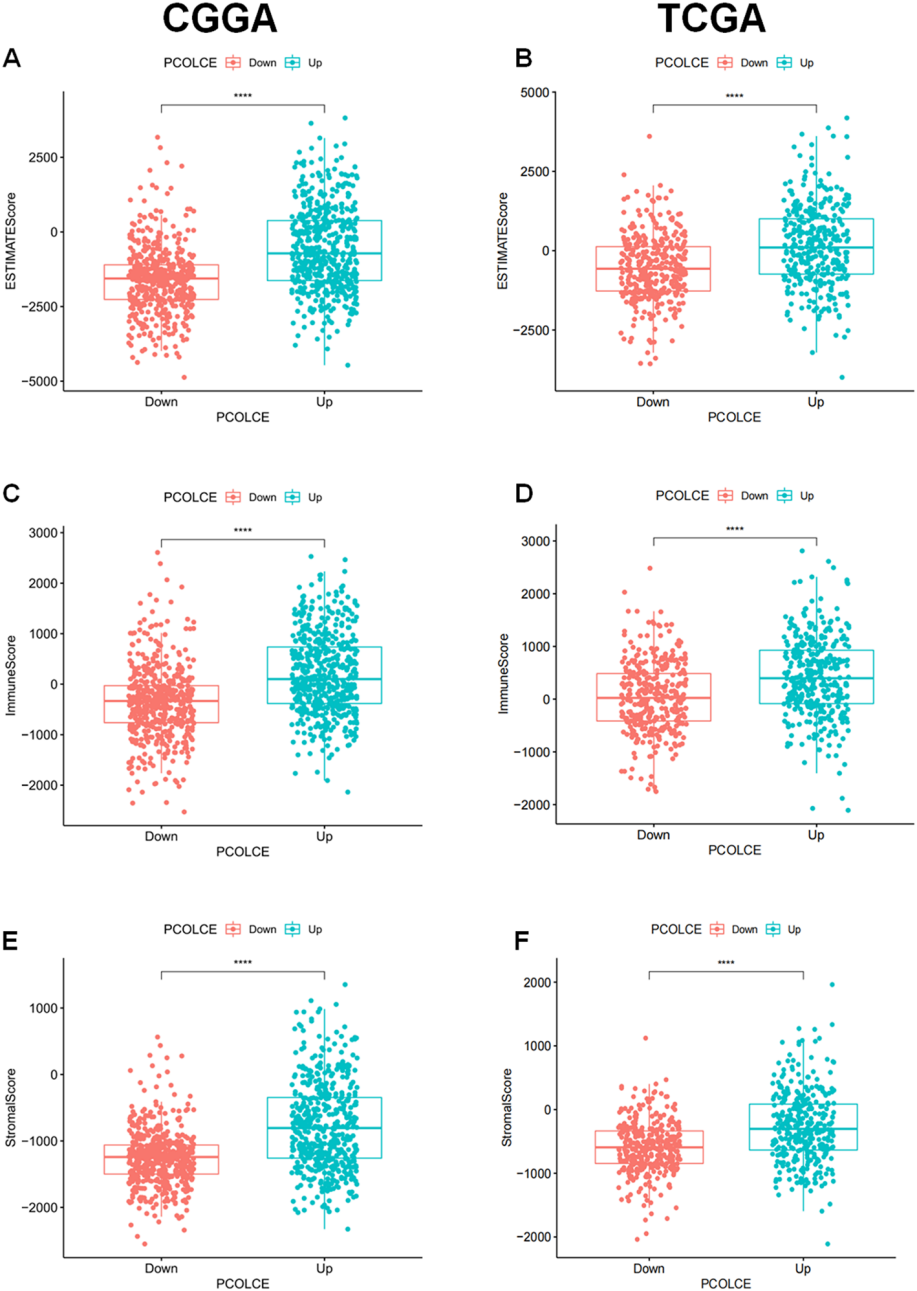
Relationship between scores and *PCOLCE* expression level in CGGA and TCGA datasets. (**A**,**B**) ESTIMATEScore, (**C**,**D**) ImmuneScore, and (**E**,**F**) StromalScore were higher in the group with higher *PCOLCE* expression. ****P < 0.0001.

**Figure 6:**
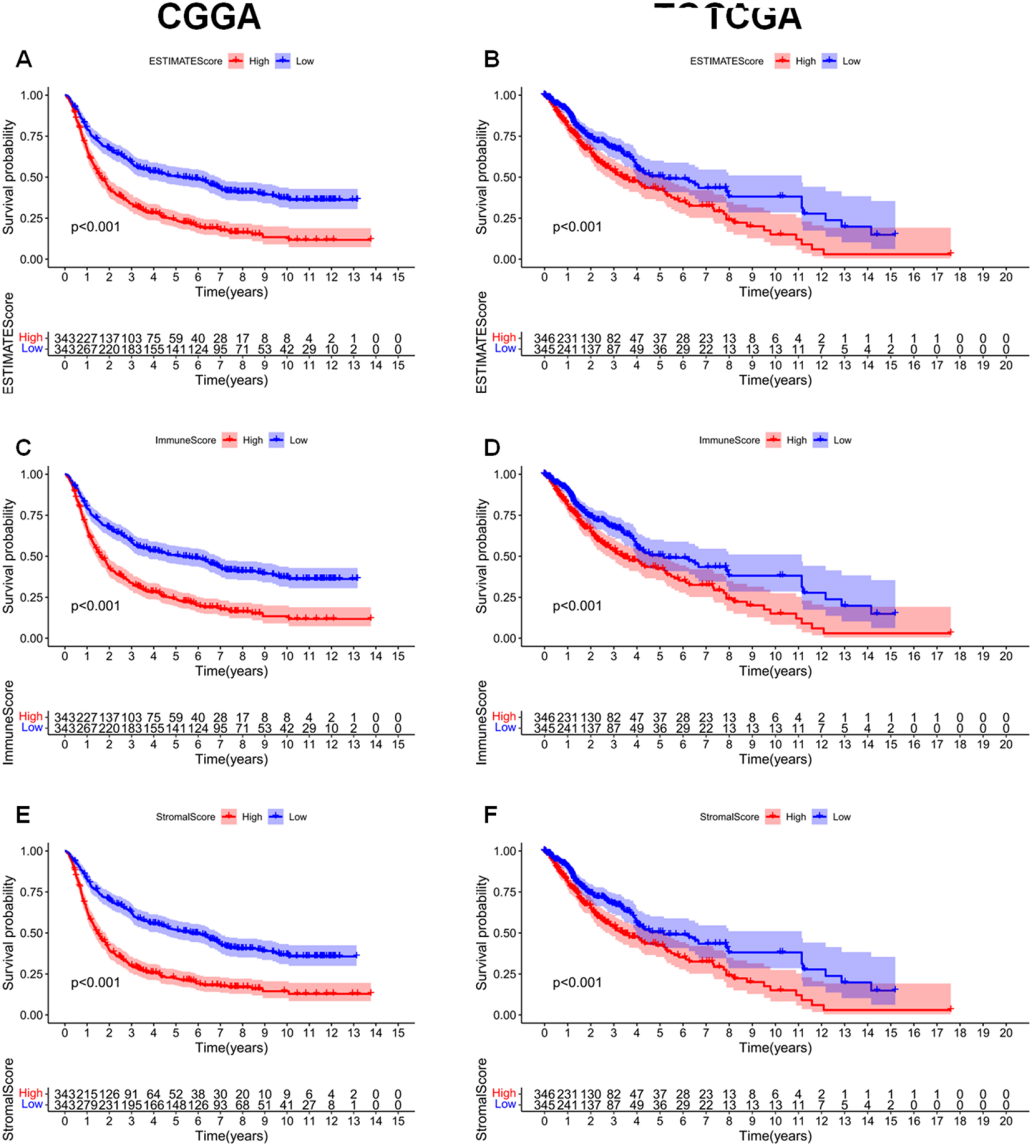
Kaplan–Meier survival curves of ESTIMATEScore, ImmuneScore, and StromalScore. High levels of (**A**,**B**) ESTIMATEScore, (**C**,**D**) ImmuneScore, and (**E**,**F**) StromalScore correlated with poor prognosis.

**Figure 7:**
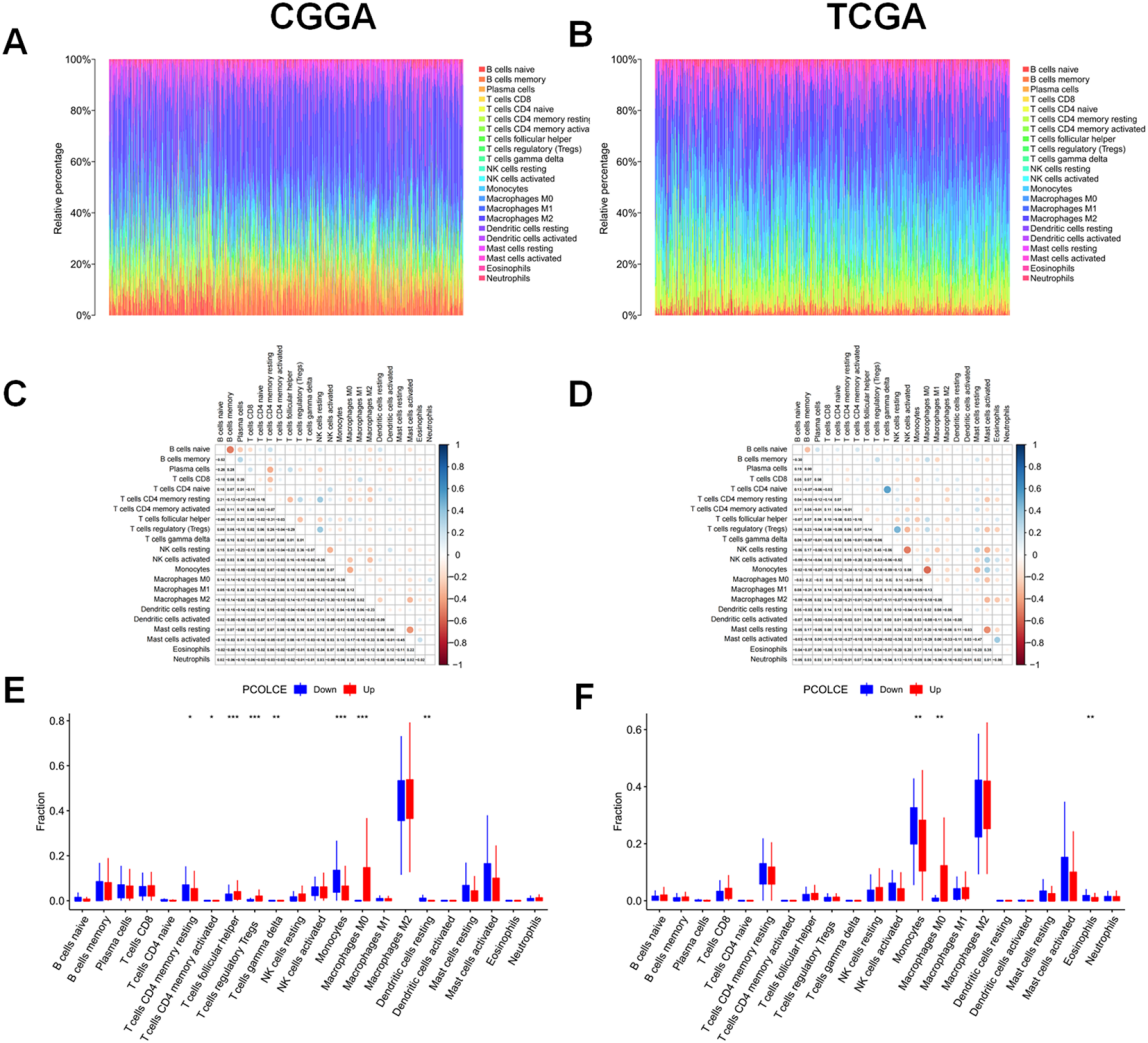
Presence and subtypes of immune cell infiltrates. The proportion of immune cells in each glioma sample is represented by a different color, and the bar lengths represent the immune cell population levels (**A**,**B**). The proportional correlation matrix of all 22 immune cells. Negatively correlated (blue); positively correlated (red). The darker the color, the higher the correlation in gliomas(**C**,**D**). Proportions of the 22 types of tumor-infiltrated immune cells in different *PCOLCE* expression groups(**E**,**F**). *P < 0.05; **P < 0.01; ***P < 0.001.

**Figure 8:**
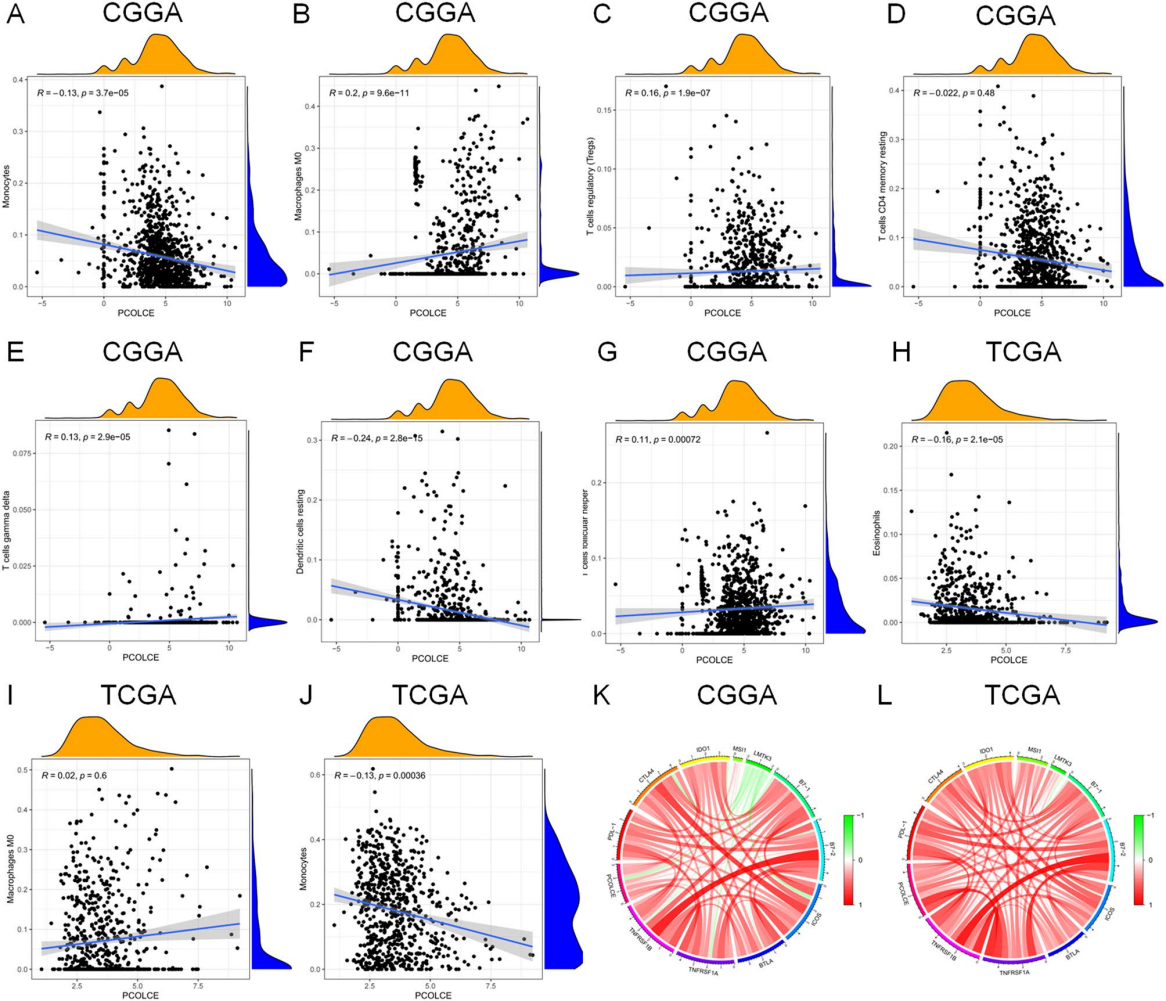
Correlation analysis between different *PCOLCE* expression groups and 22 types of immune cells in glioma using the CGGA and TCGA databases. *PCOLCE* expression was positively associated with (**B**) M0 macrophages, (**C**) regulatory T cells, (**E**) γδ T cells, (**G**) follicular helper T cells in CGGA and negatively correlated with (**A**) monocytes, (**D**) resting memory CD4^+^ T cells, and (**F**) resting dendritic cells. (**I**) M0 macrophages were positively correlated with *PCOLCE* expression in TCGA. *PCOLCE* expression was negatively associated with (**H**) monocytes and (**J**) eosinophils. The circle diagram showed that PCOLCE were positively correlated with the immune checkpoints PDL-1, CTLA-4, IDO1, MSI1, LMTK3, B7-1, B7-2, ICOS, BTLA, TNFRSF1A, and TNFRSF1B in the (**K**) CGGA and (**L**) TCGA databases.

**Figure 9:**
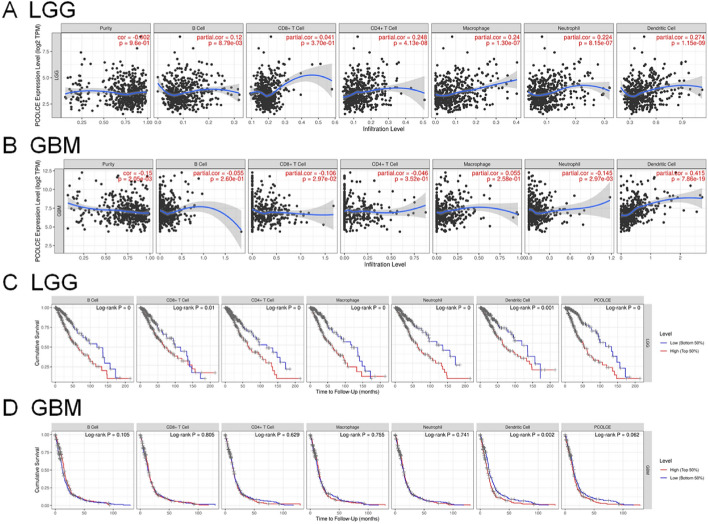
The relationship between *PCOLCE* expression level, tumor purity, and immune cell infiltration was explored using the TIMER database. *PCOLCE* was significantly correlated with immune cell infiltration in (**A**) LGG and (**B**) GBM patients. Kaplan–Meier survival analysis of several immune cells in (**C**) LGG and (**D**) GBM patients.

We used the ESTIMATE algorithm to evaluate the immune cell levels in patients with gliomas. We found significant differences in the ESTIMATEScore, ImmuneScore, and StromalScore between patients with high and low *PCOLCE* expression (P < 0.001, Fig. 5A–F). Specifically, patients with high *PCOLCE* expression had higher scores. In addition, higher scores are associated with poor prognosis of patients with glioma (P < 0.001, Fig. 6A–F). We further analyzed the correlation between PCOLCE and the 22 types of immune cells using Spearman’s correlation analysis(Fig. 7A–F). We found that PCOLCE may be related to various immune cells, including neutrophils, M1 and M2 macrophages, T cells, and other immune cells (Fig. 8A–J). We further investigated the correlation between PCOLCE and immune checkpoints and found that *PCOLCE* expression was positively correlated with several immune checkpoints: PD-L1, CTLA-4, IDO1, MSI1, LMTK3, B7-1, B7-2, ICOS, BTLA, TNFRSF1A, and TNFRSF1B (Fig. 8K,L). Finally, we used the TIMER2.0 database to explore these correlations individually in patients with LGG and GBM. The results suggested that B cells, CD8^+^ T cells, CD4^+^ T cells, macrophages, neutrophils, and DCs in LGG patients significantly affected prognosis (P < 0.05) and were associated with *PCOLCE* expression. In contrast, only DCs were associated with GBM (Fig. 9A,B). We also further analyzed the relationship between *PCOLCE* expression level, tumor purity, and immune cell infiltration and survival analysis of patients with LGG and GBM. *PCOLCE* expression was positively correlated with infiltration of CD4^+^ T cells, macrophages, neutrophils, and DCs in LGG (Fig. 9A), as well as DCs in GBM (Fig. 9B) (P < 0.01). Infiltration of CD8^+^ T cells significantly improved survival of patients with LGG (Fig. 9C), whilst B cells, CD4^+^ T cells, CD8^+^ T cells, macrophages, and neutrophils greatly survival of patients with GBM (Fig. 9D) with up regulated PCOLCE expression (P > 0.01).

### Correlation analysis between PCOLCE and related genes and immune cell markers

In addition, we subsequently performed a correlation analysis between PCOLCE and related genes, and immune cell markers (Table 1). These findings indicate that PCOLCE is associated with immune scores, checkpoints, and immune cell infiltration in patients with glioma. We also found that MS4A4A in M2 Macrophages, CCR7 in Neutrophils, and STAT6 in Th2 cells were moderately correlated with PCOLCE expression (P < 0.001; 0.40 > Cor value ≥ 0.30), whilst CD163 in M2 Macrophages, TGFB1 in Th17 cells strongly correlated with PCOLCE expression in GBM (P < 0.001; Cor value ≥ 0.40). Intriguingly, we showed that CD19 in B cells, CD163 in M2 Macrophages, HLA-DPB1, HLA-DQB1, and HLA-DRA in dendritic cells, NRP1 and TBX21 in Th1, STAT5A in Th2, PDCD1 in Th17, and GZMB in T cell exhaustion were moderately correlated with PCOLCE expression (P < 0.001; 0.40 > Cor value ≥ 0.30), whilst CD3D, CD3E and CD2 in T cells (general), HLA-DPB1 in dendritic cells, and GATA3 in Th2 strongly correlated with PCOLCE expression in LGG (P < 0.001; Cor value ≥ 0.40).

**Table 1:** Correlation analysis between PCOLCE and related genes and markers of immune cells in TIMER.

Description	Gene markers	GBM				LGG			
		None		Purity		None		Purity	
		Cor	P	Cor	P	Cor	P	Cor	P
CD8 + T cell	*CD8A*	0.226	0.005	0.208	0.015	0.184	0.000	0.196	0.000
	*CD8B*	0.057	0.486	0.005	0.958	0.095	0.030	0.083	0.071
T cell (general)	*CD3D*	0.176	0.030	0.096	0.263	0.414	0.000	0.415	0.000
	*CD3E*	0.236	0.003	0.185	0.030	0.429	0.000	0.429	0.000
	*CD2*	0.174	0.031	0.090	0.298	0.437	0.000	0.430	0.000
B cell	*CD19*	0.195	0.016	0.180	0.035	0.328	0.000	0.306	0.000
	*CD79A*	0.231	0.004	0.245	0.004	0.172	0.000	0.173	0.000
Monocyte	*CD86*	0.043	0.599	− 0.065	0.449	0.175	0.000	0.173	0.000
	*CSF1R*	0.189	0.020	0.120	0.162	0.047	0.283	0.036	0.432
TAM	*CCL2*	0.288	0.000	0.230	0.007	0.240	0.000	0.233	0.000
	*CD68*	0.243	0.003	0.189	0.027	0.276	0.181	0.277	0.716
	*IL10*	0.229	0.004	0.157	0.067	0.260	0.000	0.256	0.000
M1 Macrophage	*NOS2*	0.248	0.002	0.289	0.001	− 0.010	0.824	0.006	0.890
	*IRF5*	0.117	0.149	0.006	0.942	0.241	0.000	0.260	0.000
	*PTGS2*	0.348	0.000	0.298	0.000	0.025	0.573	0.026	0.567
M2 Macrophage	*CD163*	0.420	0.000	0.402	0.000	0.340	0.000	0.321	0.000
	*VSIG4*	0.237	0.003	0.171	0.045	0.101	0.021	0.089	0.051
	*MS4A4A*	0.326	0.000	0.303	0.000	0.248	0.000	0.241	0.000
Neutrophil	*CEACAM8*	0.074	0.366	0.084	0.331	0.094	0.032	0.091	0.046
	*ITGAM*	0.241	0.003	0.187	0.028	0.163	0.000	0.172	0.000
	*CCR7*	0.327	0.000	0.302	0.000	0.283	0.000	0.281	0.000
Natural killer cell	*KIR2DL1*	0.126	0.121	0.118	0.171	0.134	0.002	0.128	0.005
	*KIR2DL3*	0.104	0.000	0.081	0.349	0.217	0.000	0.213	0.000
	*KIR2DL4*	0.057	0.482	0.033	0.706	0.178	0.000	0.172	0.000
	*KIR3DL1*	0.230	0.004	0.221	0.010	0.083	0.060	0.073	0.111
	*KIR3DL2*	0.116	0.154	0.119	0.165	0.104	0.018	0.109	0.017
	*KIR3DL3*	0.124	0.126	0.144	0.094	0.001	0.985	0.001	0.980
	*KIR2DS4*	0.263	0.001	0.272	0.001	0.177	0.000	0.177	0.000
Dendritic cell	*HLA-DPB1*	0.222	0.006	0.180	0.035	0.414	0.000	0.407	0.000
	*HLA-DQB1*	0.150	0.064	0.133	0.122	0.355	0.000	0.334	0.000
	*HLA-DRA*	0.185	0.023	0.111	0.195	0.398	0.000	0.390	0.000
	*HLA-DPA1*	0.168	0.038	0.129	0.134	0.396	0.000	0.386	0.000
	*CD1C*	0.160	0.048	0.084	0.329	0.225	0.000	0.201	0.000
Th1	*NRP1*	0.553	0.000	0.551	0.000	0.329	0.000	0.310	0.000
	*ITGAX*	0.044	0.587	− 0.029	0.736	0.210	0.000	0.220	0.000
	*TBX21*	0.176	0.029	0.181	0.034	0.370	0.000	0.360	0.000
	*STAT1*	− 0.113	0.165	− 0.107	0.213	0.276	0.168	0.266	0.000
	*IFNG*	0.048	0.557	0.038	0.663	0.209	0.000	0.202	0.000
	*TNF*	0.099	0.223	0.017	0.844	0.002	0.957	− 0.009	0.839
Th2	*GATA3*	0.256	0.001	0.239	0.005	0.438	0.000	0.442	0.000
	*STAT6*	0.358	0.000	0.310	0.000	0.125	0.004	0.156	0.001
	*STAT5A*	0.195	0.016	0.158	0.065	0.306	0.000	0.320	0.000
	*IL13*	0.016	0.842	0.029	0.734	0.008	0.848	0.030	0.514
Tfh	*BCL6*	0.006	0.943	0.015	0.862	− 0.050	0.262	− 0.060	0.193
	*IL21*	− 0.027	0.745	0.010	0.908	0.053	0.228	0.044	0.340
Th17	*STAT3*	0.150	0.064	0.161	0.060	0.277	0.000	0.253	0.000
	*IL17A*	0.029	0.719	0.008	0.925	0.097	0.027	0.083	0.069
	*FOXP3*	0.143	0.077	− 0.157	0.067	0.002	0.960	0.015	0.740
	*CCR8*	0.269	0.001	0.230	0.007	0.189	0.000	0.202	0.000
	*STAT5B*	0.011	0.892	0.067	0.435	− 0.035	0.430	− 0.031	0.501
	*TGFB1*	0.457	0.000	0.407	0.000	0.306	0.000	0.307	0.000
T cell exhaustion	*PDCD1*	0.222	0.006	0.199	0.020	0.378	0.000	0.370	0.000
	*CTLA4*	0.180	0.026	0.131	0.128	0.242	0.000	0.218	0.000
	*LAG3*	0.039	0.635	0.080	0.354	0.277	0.000	0.272	0.000
	*HAVCR2*	0.053	0.516	− 0.074	0.392	0.209	0.000	0.215	0.000
	*GZMB*	0.186	0.021	0.139	0.106	0.346	0.000	0.347	0.000

### Analysis of immunophenoscore (IPS) and the sensitivity of multi-agents

**Figure 10:**
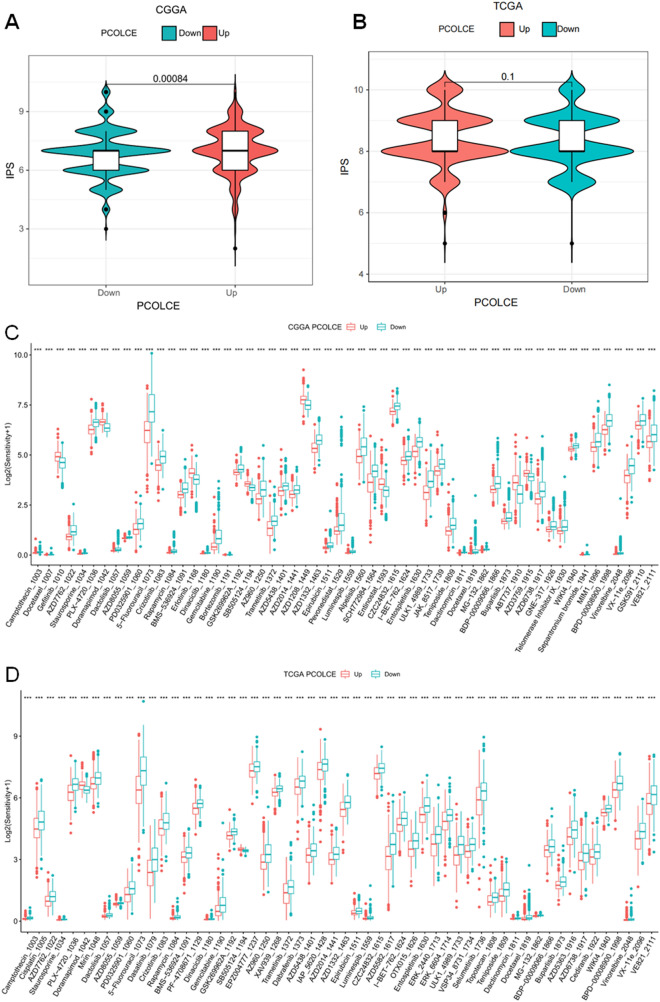
IPS score and drug sensitivity analysis of differential PCOLCE expression in CGGA and TCGA patients. (**A**,**B**) IPS score in the high and low PCOLCE expression groups. (**C**,**D**) Drug Sensitivity in the high and low PCOLCE expression groups.

PCOLCE expression was higher in gliomas with higher IPS Z-scores in CGGA (P < 0.001). Although TCGA showed a similar trend, it was not statistically significant (P = 0.1) (Fig. 10A,B). glioma cells with high expression of PCOLCE were sensitive to Gefitinib_1010, Doramapimod_1042, Erlotinib_1168, AZD1208_1449, ABT737_1910 and AZD3759_1915 in CGGA (P < 0.001), as well as Doramapimod_1042 and SB505124_1194 in TCGA (P < 0.001, Fig. 10C,D).

## Discussion

*PCOLCE* is involved in the progression of many types of cancers; however, its role in glioma was unknown. Based on analyses of TCGA and GTEx databases, We found that PCOLCE was more highly expressed in glioma tissue than in normal brain tissue, especially in GBM. Similarly, HPA results also showed a high level of PCOLCE in glioma. These results suggest that PCOLCE may play a role in exacerbating glioma progression.

Further analysis revealed that the higher the WHO grade of glioma, the higher the expression of PCOLCE. Similarly, high PCOLCE expression was associated with high wild-type IDH1 level, non-methylated MGMT and incomplete 1p19q deletion compared with the low expression group. Previous studies have shown that IDH mutations led to better clinical outcomes and a longer median 2-year survival in GBM (IDH wild-type: 15 months; IDH mutant: 31 months) and anaplastic astrocytoma (IDH wild-type: 20 months; IDH mutant: 65 months)^27^. Interestingly, IDH mutations occurred in 73% of clinical cases in secondary GBM and were rare in primary GBM (3.7%)^28^. The high incidence of IDH mutations in secondary GBM is associated with the frequent relapse of IDH mutations in low-grade gliomas^29^. Compared with young patients with WHO grade II/III glioma, patients with primary GBM rarely show this mutation^30^. Therefore, we hypothesized that the high *PCOLCE* expression in primary GBM patients might be related to IDH wild-type tumors.

Kaplan–Meier curve survival analysis showed that high expression of *PCOLCE* significantly affects the prognosis of glioma patients.To confirm this result, univariate and multivariate Cox analyses were performed. Based on these findings, high expression of PCOLCE was identified as an independent prognostic factor for patients with gliomas. To further confirm our results, ROC curve analysis was performed, which also showed satisfactory performance of PCOLCE for 1-, 3- and 5-year OS prediction.

To identify the mechanisms by which high expression of *PCOLCE* affects the survival of patients with glioma, GO and KEGG enrichment analyses were performed using different *PCOLCE* expression levels. The results showed that multiple immune-related pathways were enriched, which was consistent with the results of GSEA. The above three analyses not only confirmed that PCOLCE may act by regulating tumor immune interactions, but also provided new findings, such as the involvement of neutrophils, T cell activation, and B cell-mediated immunity in the immune response of glioma cells, which have not been identified in previous studies.

There was significantly positive correlation between high expression of PCOLCE and high ESTIMATEScore, ImmuneScore, and StromalScore in patients with glioma. All of which are associated with poor patient prognosis. This association further indicates that *PCOLCE* may interact with the immune-related TME. Further analysis confirmed that the proportion of immune cells was different among glioma samples and significantly differed between PCOLCE groups. In addition, *PCOLCE* expression was positively correlated with multiple immune checkpoints. Studies have shown that the immune checkpoint ligands ICOS, BTLA, TNFRSF1A, and TNFRSF1B all contribute to glioma immune evasion^31,32^. We also assessed the ratios of different immune cells using CIBERSORT and found significant differences between the different *PCOLCE* expression groups. Among them, monocytes, M0 macrophages, and Tregs were positively associated with *PCOLCE* expression, which further supported tumor-immune associations. Monocytes reside in the bone marrow, blood, and spleen of vertebrates but can be recruited to infected or injured tissues to act as effector cells, especially progenitor DCs and macrophages^33^. Previous research indicates that glioma recurrence decreases the number of invading monocytes and increases glioma-associated macrophages/microglia (GAMPs)^34^. GAMPs, as tumor-supporting cells, have been shown to promote glioma growth and invasion^35^. Due to the significant negative correlation between M0 macrophages, monocytes, and OS, we speculate that M0 macrophages play an important role in glioma development after monocyte transformation. Another important finding was that *PCOLCE* expression was associated with multiple levels of immune infiltration in gliomas. They were significantly correlated with dendritic cells, CD4^+^ T cells, macrophages, neutrophils, and B cells in LGG; high *PCOLCE* expression was also correlated with dendritic cells and GBM purity.

Our study found that high PCOLCE expression is associated with poor prognosis in patients with glioma, which may be due to suppression of tumor-associated immune cells. Additionally, some of these immune cells in patients with LGG influence prognosis and correlate with PCOLCE expression. However, these same results were not found in GBM, suggesting differences in PCOLCE function between LGG and GBM. Therefore, further mechanistic studies are required.

However, glioma tumors are highly heterogenous and include not only the glioma cells, but also the glioma-associated non-neoplastic cells, such as stromal cells and immune cells^36^.Although these non-tumor cells, particularly immune cells, act as pivotal players in glioma progression, the presence of these cells dilutes the purity of glioma cells^37,38^. Tumor and non-tumor cells cooperate with each other to maintain the delicate homeostasis of glioma initiation, malignant tumor progression, and therapeutic resistance^39,40^.Therefore, after adjusting the tumor purity, the results still showed that the expression level of PCOLCE in GBM and LGG tissues was closely related to most immune cell markers (Table 1).

To predict cytotoxic T lymphocyte antigen-4 (CTLA-4) and anti-PD-1 antibody responses to immune checkpoint blockades (ICB), immunophenoscore (IPS) assays were performed^41^. Generally, there was a positive correlation between IPS and ICB responses. However, We found that PCOLCE expression was higher in glioma patients with high IPS z score in CGGA, and TCGA showed a similar trend, but not significant. This may also partly explain there have been no breakthroughs in targeting CTLA-4 and anti-PD-1 in the treatment of glioma.

The sensitivity of multi-agents was also calculated utilizing an R package (oncoPredict package)^42^ to explore potential chemotherapeutic agents for targeting up—and downregulated PCOLCE expression. High expression of PCOLCE is resistant to most chemotherapy drugs, which is consistent with our expectation. However, We first found that glioma cells with high PCOLCE expression were sensitive to multiple chemotherapeutic small molecules in CGGA and only two in TCGA. Therefore, for future chemotherapy and drug development for treatment of gliomas with high PCOLCE expression, these drugs may be promising candidates.

However, our study has some limitations. We did not investigate the mechanisms through which PCOLCE influences immune function in gliomas. In addition, we did not experimentally validate the correlations between PCOLCE and the TME. Lastly, we did not investigate the role of PCOLCE in glioma subtypes, such as diffused midline and recurrent gliomas.

In conclusion, here we analyzed glioma data from TCGA and CGGA databases simultaneously, which produced consistent outcomes, thus supporting our results. These results suggest that PCOLCE significantly influences the prognosis of patients with glioma, can serve as an independent prognostic factor, and is associated with tumor immunity. PCOLCE may be a novel immune-related target for treating gliomas. Additionally, analysis of chemosensitivity in patient gliomas with high PCOLCE expression may provide a promising direction for drug development. This gene has rarely been reported in previous glioma studies, therefore, our research may provide useful information for future research direction, new diagnosis and treatment targets for glioma.

## Supplementary Information

Supplementary Information 1.

Supplementary Information 2.

## Data Availability

Public data available within this article can be found at: http://www.tcga.org, http://www.cgga.org.cn.
