# Supplementary material for: High expression of PCOLCE gene indicate poor prognosis in patients and are associated with immune infiltration in glioma
Source: Sci Rep. 2023 Mar 7;13:3820. doi: 10.1038/s41598-023-30413-5 (PMC9992371; doi:10.1038/s41598-023-30413-5)
Supplement: Supplementary file 2 — Supplementary Information 2. [file 41598_2023_30413_MOESM2_ESM.docx]

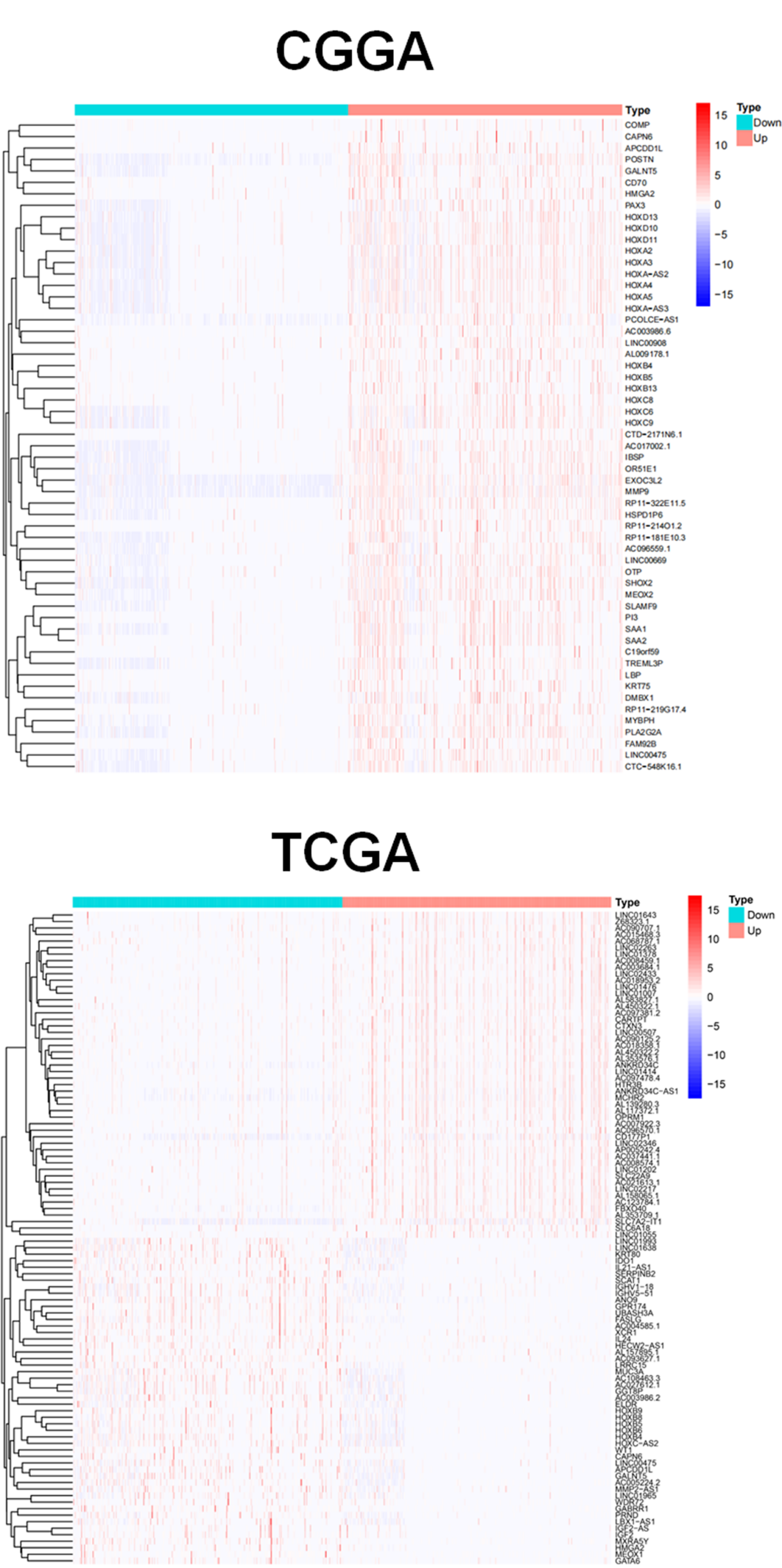


**SUPPLEMENTARY FIGURE 2**| The differential genes and the heatmap based on the expression level of *PCOLCE* in glioma to display the top 50 upregulated and downregulated genes
